# Emotional dysregulation and linguistic patterns as a defining feature of patients in the acute phase of anorexia nervosa

**DOI:** 10.1007/s40519-022-01456-w

**Published:** 2022-08-08

**Authors:** Rachele Mariani, Isabella Marini, Michela Di Trani, Carlotta Catena, Francesca Patino, Raffaele Riccioni, Massimo Pasquini

**Affiliations:** 1 Department of Dynamic and Clinical Psychology, and Health Studies, Sapienza University of Rome, Rome, Italy; 2 Azienda Ospedaliera-Universitaria, Policlinico Umberto I, Rome, Italy; 3 Department of Human Neurosciences, Sapienza University, Rome, Italy

**Keywords:** Anorexia nervosa, Emotional regulation, Linguistic analysis, Alexithymia, Autobiographical narratives

## Abstract

**Purpose:**

This research aims to analyze the relationship between emotional regulation and the symbolic process in autobiographical narratives of a group of individuals diagnosed with restrictive anorexia nervosa (AN), compared to a non-clinical group. The study is framed within multiple code theory (MCT) (Bucci, 1997; 2021), which considers mind–body integration. The purposes of this study are to investigate whether participants of the AN group will show greater alexithymia and emotional dysregulation than the non-clinical group; and whether the specific linguistic and symbolic features, such as somato-sensory words, affect words, and difficulty in the symbolizing process will predict the AN group.

**Methods:**

Twenty-nine female participants hospitalized with AN during an acute phase (mean age 19.8 ± 4.1) and 36 non-clinical female participants (mean age 21 ± 2.4) were selected through snow-ball sampling. The participants completed the Toronto Alexithymia Scale (TAS-20), the Profile of Mood of State (POMS), the Emotion Regulation Questionnaire (ERQ), and the Relationship Anecdotes Paradigm Interview (RAP). The RAP interview was audio-recorded and transcribed to apply the Referential Process (RP) Linguistic Measures. A *T* test for paired samples and a logistic binary regression was performed.

**Results:**

AN presented a significantly higher emotional dysregulation through the ERQ, TAS20 and POMS measures. Specifically, AN showed higher ER expression/suppression strategies, fewer functional cognitive strategies, higher alexithymia, and higher mood dysregulation. Specific linguistic features such as sensory-somatic, word affect, and difficulty in RP symbolizing predict the AN group (R2 = 0.349; *χ*2 = 27,929; df = 3; *p* = .001).

**Conclusions:**

Emotional dysregulation is connected to AN symptoms and autobiographical narratives. The results can help a clinical assessment phase showing specific linguistic features in AN patients.

**Level of evidence:**

Level II, controlled trial without randomization.

## Introduction

### Clinical symptom classification AN

Anorexia nervosa (AN) is a psychiatric syndrome characterized by a reduction in calorie intake, followed by a pathological decrease in body weight. Anorexia is a condition of self-induced food restriction, accompanied by a deep desire to be thin, and/or fear of fatness. which involves the appearance of symptoms and clinical signs related to the conspicuous weight loss.

AN is associated with significant psychological and relational impairment in the person and is one of the main contributors to the reduction of the quality of life in the context of eating disorders. Affected patients show a lower health-related quality of life (HRQoL) than the healthy population [1]. In Western countries, including Italy, it is estimated that the prevalence of anorexia nervosa is around 0.2–0.8% and that the incidence is 4–8 new cases per year, per 100,000 individuals [2]. The average age of onset is between 14 and 18 years and the diagnosis is ten to twenty times more frequent in women than in men.

There are two different clinical manifestations of anorexia nervosa: the restrictive type I, in which weight loss is mainly achieved through a restrictive diet, fasting, and sometimes excessive physical activity; and type II, with the accompaniment of binging/purging behaviors. The course of AN is variable, and while spontaneous recovery without treatment is possible, a fluctuating course is more likely the case in which the affected individual shows signs of improvement while remaining anchored to some of the preoccupations regarding food, weight, and bodily appearance. Related studies show conflicting evidence about the severity of the two different types. In fact, some research argues that type I is associated with lower chances of recovery, compared to those with the variant consisting of binges and eliminatory behaviors, notwithstanding its association with a lower suicide rate [3]. Other studies report that binge–purge presentations fare off worse [4]. Finally, the high crossover rate in the sample from type I to type II suggests that restrictive AN could be considered a phase in the course of AN rather than a distinct subtype [5]. The advantage after hospitalization is undoubtedly in terms of weight, but it is less concrete in terms of socialization and mood; poor relations with peers remain, and sometimes severe depressions are highlighted [6]. Quantifying the mortality rate of anorexia nervosa is a complex matter. Some studies indicate a mortality rate of approximately 5.25% and a Standardized Mortality Ratio (SMR) of 9.7 [7]. The SMR of anorexia nervosa is by far one of the highest in the context of psychiatric disorders.

The severity of the disorder and the implications related to high mortality show the need to analyze assessment factors that can help better understand the psychological factors underlying the symptomatology of AN. One aspect of AN symptom severity is body mass index (BMI) [8]. Mild severity is attributed for a BMI > 17 kg/m2, moderate for a BMI between 16 and 16.99 kg/m2, severe if the BMI is between 15 and 15.99 kg/m2, and extreme for a BMI < 15. Despite the clear ease and convenience with which the BMI is used to define the severity of the anorexic syndrome, an exclusive reference to the numerical value seems reductionistic and simplistic. The clinical history predicts a gradual decline and physical deterioration, leading to the deadly consequences of starvation. In addition to an evaluation of the entity of weight loss, it is necessary to reflect on the more general implications related to it, such as the influence that this has on body related attitudes (BodyRA) and especially, on body dissatisfaction in adolescents [9].

### Emotional regulation and alexithymia in AN

Several studies have emphasized the relationship between AN and the individual’s deficits in emotional awareness [10–12], or heightened alexithymia. Alexithymia can be defined as quite literally, ‘a lack of words to express emotion,’ [13] characterized by difficulties in identifying and describing emotions, as well as employing fantasy to regulate painful feelings and finding creative solutions to problems, and communicating needs to others to obtain support [14]. The fight against the feeling of hunger and the long abstinence from it seems to lead to a greater reduction in feeling emotion and related facial expressions, and showing higher alexithymia in the acute phase, compared to that which occurs in recovered patients [15].

Individuals with AN also display alexithymia, closely related to the difficulties in identifying feelings about themselves [16, 17]. Foye et al. [18] pointed out that AN represents an attempt to modulate current or persistent aversive emotional arousal, in a dysfunctional way. Avoiding emotions could be considered to be a coping strategy against feeling affect, avoiding positive or negative emotions, showing poor interoceptive awareness, and displaying confusion of emotional states and difficulties with emotional language. Recent review findings even suggest that although overall empathy levels may be intact in AN, greater emotional regulation difficulties, elevated levels of alexithymia, and reduced emotional awareness can still be evident [19]. Various empirical studies reveal that emotional regulation plays an important role in the development, maintenance, and psychopathology of AN [20–23]. Research that used the Emotional Regulation construct confirmed that AN is characterized by dysfunctional emotional regulation strategies oriented towards a suppressive strategy rather than cognitive re-evaluation [24].

### AN according to the multiple code theory

Multiple code theory (MCT) is a general theory of emotional information processing, derived from current work in cognitive psychology, psychoanalysis, and affective neuroscience [25]. Taylor and Bagby [26] suggested that alexithymia may be more fully understood in light of Bucci’s [27] multiple code theory (MCT).

This model poses that information is processed in parallel [28] among three different systems, the subsymbolic, non-verbal symbolic and verbal symbolic systems, which organize the experience in a global and integrated way. Bucci [29] argues that a Referential Process (RP) connects these three types of processing systems and allows people to express their emotional experiences into words. In other words, Alexithymia can be further understood as an expression of the disconnection of RP [30]. The RP and emotional communication are connected to Damasio’s notion of dispositional representations [31] that provide a neurobiological basis for the concept of emotional schema. These are complex units that combine emotion and cognition, necessarily founded and rooted in bodily interactions with others [32]. More specifically, they are made up of desires, expectations, and beliefs about other people that develop during interactions with the latter, from the beginning of life. Consequently, the individuals and situations in which these relationships took place constitute the specific contexts and contents of the emotion patterns [32]. The latter, therefore, includes patterns of visceral or somatic experience associated with the emotional experience, as well as images of the object of emotion or what we desire or hate and fear. These innate dispositional representations can be associated to the arousal/emotional activation and to a non-conscious state of the self, where sub-symbolic systems and bodily experiences are predominant. The dispositional representation is activated in autobiographical memories, which can activate emotional schemas [31]. A good RP allows for the representation of emotional schema memories in verbal forms, which follows the implications of MCT [32], concerning access to the experiential and preverbal code of the implicit self [33, 34]. The food restriction and procedures that AN describes as connected to the autobiographical narratives of individuals assumes the function of an organizational symbol within emotional schemes. Nandrino et al. [35] showed that AN is characterized by an excessive generalization of autobiographical memories that would lead individuals to manifest greater difficulties in making use of an affective narrative, capable of leading the other to "live" their own experience. In addition, patients with AN tend to use specific cognitive and behavioral strategies to avoid or mitigate negative affect [36]. The verbalization process can represent a measure of the capacity to emotionally regulate, which is expressed through linguistic indicators. In Mariani et al. [37], what was found to emerge was a representative linguistic pattern of emotional regulation difficulties in narrative processes in psychopathology, such as sensory-somatic words and the presence of strong negative feelings.

We hypothesize that according to MCT, a specific pattern of emotional regulation in AN patients will be seen in both, emotional regulation strategies, as well as in linguistic patterns in autobiographical narratives. More specifically:

- Individuals diagnosed with AN will show greater levels of alexithymia, emotional dysregulation, and affect dysregulation compared to non-clinical groups;
- Specific linguistic features, that show difficulties in the symbolization process, such as higher sensory-somatic words, higher negative affect words, and dysfunctional RP patterns will predict the clinical group.

## Method

### Participants

A clinical sample of patients seeking hospitalization or visiting an inpatient unit at a day hospital was recruited for the study. The diagnosis of restrictive anorexia nervosa was confirmed using the semi-structured interview, SCID-5-CV [38]. The constitution of the clinical group was made according to the following criteria: Inclusion: between 16 and 30 years of age, experiencing an acute phase of AN, BMI ≥ 12.5 kg/m^2^, Time from diagnosis ≥ 6 months; and Exclusion: Chronic pathology (time from diagnosis ≥ 10 years), diagnosis of acute psychosis, mental retardation, cognitive impairment, ongoing alcohol or psychoactive substance abuse and/or other conditions that may affect the understanding of the questionnaires and the ability to provide informed consent. 31 female clinical participants were recruited, of which 29 agreed to participate in the study. As a result, our clinical sample consists of 29 participants, mean age 19.8, sd. 4.1; year of education 12.2, s.d. 2.3; patients were classified according to the severity level of BMI at time of study (Table 1). Simultaneously, a control group was recruited, pairing for age and educational attainment. The exclusion criteria for the control group was the presence of major mental disorder and eating disorder symptoms, which was evaluated using the Eating Attitudes Scale test (EAT-26) [39]. 36 non-clinical female participants have been included, mean age 21, sd 2.4; year of education 12.6, s.d. 2.5. The non-clinical sample’s EAT-26 mean was 9.04, sd. 4.09; 11 participants have been excluded from the study due to the EAT-26 clinical cutoff (> 26). All non-clinical participants reported being in good health and had an average BMI (between 19 and 24). Other main characteristics are shown below (see Table 1).

**Table 1:** Socio-demographics and clinical characteristics of the sample group

	AN Group (N. 29)		Control Group (N. 36)	
	*M*	*Sd*	*M*	*Sd*
Age (yy)	19.8	4.1	21	2.4
Education (yy)	12.2	2.3	12.6	2.5
BMI (Kg/m2)	16.14	1.39	22.26	1.74
Eating Disorder Severity (BMI)	**%**			
Mild AN (BMI > 17)	41		–	–
Moderate AN (16.99 > BMI > 16)	21		–	–
Severe AN (15.99 > BMI > 15)	10		–	–
(Extreme AN (BMI < 15)	26		–	–
Eating Disorder Severity (EDI-3)	*M*	*Sd*		
EDRC	73	26	–	–
GPMC	77	22	–	–
Duration of illness (mm)	10.17	2.4	–	–
Length of treatment (mm)	8.2	1	–	–
Type of treatment	%		%	
Psychotherapy	20.69		13.9	
Psycopharmacotherapy	27.59		5.6	
Integrated treatment	51.72		0.0	

The sample numerosity has been estimated by G*Power 3.1.9.2 (Düsseldorf, Germany) for the two groups comparison on an effect size of f 0.25 (mean), power of 80%, assuming α 0.05.

### Procedure

Following the recruitment procedure, described above, participants completed the informed consent. Research involved the administration of standardized self-report tests through a special form and, subsequently, of an interview through online platforms, due to the COVID-19 pandemic. The interview was carried out through an online meeting program and The Relational Anecdotical Protocol method of interviewing was used. This method of interviewing is similar to that of a psychotherapy session transcript analysis, which offers a broad scope of studying the narratives [40, 41]. The administration of the measures took place in similar settings for all groups individually, with a qualified and experienced psychologist. The audio-recorded interviews were transcribed following specific procedures for the Italian version of the Discourse Attributes Analysis Program (DAAP) [42] for the computerized application of the RP linguistic measures. The protocol has been approved by the Department Ethics Committee, where the study was developed.

### Measures

The research consisted of different questionnaires, including the following:

- *Socio-demographic questionnaire—*a demographic sheet that contains questions regarding gender, age, level of education, marital status, family of origin, current occupation, and whether the individual is currently in psychotherapy and/or receiving pharmacological treatment;
- *Profile of Mood of State*: POMS [43, 44]—a self-report psychological measure of mood states consisting of a 65-item mood adjective checklist in which each adjective is scored from 0 (absent) to 4 (very much), based on how well each item describes the respondent's mood during a specified time frame (during the past week, including today). Following the standard scoring method for the Italian population, six mood scales are derived: tension–anxiety, depression–dejection, anger–hostility, vigor activity, fatigue–inertia, and confusion–bewilderment. A higher score denotes a higher level of a certain emotion. In the current study, Cronbach's alpha was found to vary between 0.82 and 0.91.
- *Toronto Alexithymia Scale*: TAS-20 [45]—a questionnaire that evaluates the presence of clinical or non-clinical alexithymia, and, therefore, identifies the subjects most at risk of pathology. Three factors emerge from the factor analysis, which correspond closely to the construct: Difficulty in identifying feelings (DIS) and distinguishing between emotions and bodily sensations; and Difficulty in describing feelings (DDS); The TAS-20 uses cutoff scoring: equal to or less than 51 = non-alexithymia, equal to or greater than 61 = alexithymia. Scores of 52 to 60 = possible alexithymia. In the current study, Cronbach’s alpha value was 0.81
- *Emotion Regulation Questionnaire*: ERQ [46, 47]—a self-report questionnaire that includes 10 items and contains two scales corresponding to two different emotional regulation strategies: 1. Reappraisal (6 items): cognitive restructuring, a conscious mental process that allows to modify the interpretation associated with an emotional stimulus. Higher scores in this factor show a good strategy to reduce stressful effect (Gross, 2002); 2. Suppression (4 items): an attempt to suppress the expression of emotions through both verbal and body language, higher scoring in this factor shows emotional distress. For the present study, Cronbach’s alpha was found to vary between 0.76 and 0.85.
- *Linguistic measures of the Referential Process*: (see Table 2)
- Computerized language measures have been applied to the transcripts of the interviews of the referential process, according to the multiple code theory [25], using Italian Discourse Attributes Analysis Program (IDAAP) software [48; 49]. Listed below are the linguistic measures:
- *Italian Weighted Referential Activity Dictionary* IWRAD [48]—a list of 9596 frequently used linguistic elements. The dictionary contains words with a high-frequency function, such as articles, pronouns, and prepositions that are aspects of the generative function of language. IWRAD evaluates language style rather than content, represents unintended aspects of emotional expression, and provides microanalytic tracking of the Symbolizing phase of RP. For a deeper discussion on the method of building a weighted dictionary, such as IWRAD, see [49].
- The *Italian Mean High Weighted Referential Activity Dictionary* (MH-IWRAD)—a derivate variable of IWRAD, which is defined as the Referential Activity Intensity Index. This is essentially a measure of high intensity emotional engagement emerging from speech [48]. It is obtained by counting IWRAD scores lying above the neutral value. This is perhaps best understood as a measure pick of IWRAD.
- *The Italian DisFluency Dictionary* (IDFD)—a small set of 11 words and repeated words, incomplete words, and filled pauses that people tend to use when struggling to communicate [50]. A score on this index corresponds to the proportion of IDFD words present in the speech. High scores typically characterize the arousal phase in which emotional schemas are activating.
- *Italian Reflection Dictionary* (IREF) [48]—a dictionary that contains words about the way people think and communicate thoughts. It includes basic logical words and elements that refer to cognitive, logical, or impaired functions. These elements will probably dominate in the reorganization phase.
- The *Italian Sensory Somatic Dictionary* (ISensD)—a list of Italian words related to the body and bodily activities, and to sensory processes and/or descriptions of symptoms [29]. The number of ISensD words in a speech sample is a measure of the arousal of bodily, sub-symbolic aspects of emotion schemas.
- The *Italian Sum Affect Dictionary* (ISAffD) [48]—contains Italian words concerning how people feel and communicate feelings directly. It includes emotion labels and functions associated with affective arousal. ISAffD consists of three sub-dictionaries related to domains of affect: positive affect (IPAffD), negative affect (INAffD), neutral affect without a specific valence (IZAffD).
- The *relationship anecdotal paradigm*: (RAP) [51] Interviewees are presented with the following prompt, “Please tell me some incidents or events about an interaction between yourself in relation to another person” (Luborsky, 1998, p. 110), specifying when it occurred, with whom it occurred, something about what the other person said or did, and what happened in the end. Interviewees are asked to tell between 6 to 10 relationship episodes and are free to describe any incidents involving any person. Evidence for the validity of the RAP was provided by Barber, Luborsky, Crits-Christoph, & Diguer [52].

**Table 2:** Examples of words belonging to Italian linguistic measures of the Referential Process

IWRAD high weight: odore (smell = 0.981); maledetto (damned = 0.890); urla (scream = 0.835); stupore (astonishment = 0.786); baciare (to kiss = 0.747);
IWRAD medium weight: il (the = 0.058); capisci (you understand = 0.0314); io (I = 0.018); lei (she = 0.008), ciò (that = −0.011); tu (you = −0.042)
IWRAD low weight: superficiali (superficial = −0.870); parlavamo (we talked = −0.870); ansiosa (anxious = −0.867); capiscono (they understand = −0.654); carini (nice = −0.519)
IRefD: Attenzione (attention), capire (to understand), decisione (decision), dubbio (doubt), meditare (to meditate), ragione (reason), razionalità (rationality), ricordo (memory)
IDFD: Quindi (so), cioè (that is), comunque (however), allora (then), insomma (well), niente (nothing), magari (maybe), vabbè (don’t care), boh (don’t know), and ‘ehmm’ and ‘mm’ representing filled pauses with slightly different significance
ISenSD: Ammalato (sick), digerire (digest), disorientamento (disorientation), dolore (pain), impotente (impotent), innervosito (unnerved), pesare (weigh), ridere (laugh), sintomi (symptoms), vomitare (throw up)
INAffD: Abbandonato (abandoned), depresso (depressed), impaurito (frightened), invidioso (envious), malinconia (gloom), odio (hate), sofferenza(suffering)
IPAffD: Abbracci (hugs), affidabile (reliable), baciare (to kiss), felice (happy), innamorato (in love), speranza (hope)
IZAffD: Attesa (expectation), bisogno (need), coinvolto (involved), eccitato (excited) intensità (intensity), motivazione (motivation), reagire (react), sensazione (feeling)
ISAffD: Sum of all Positive, Negative, Neutral Affect Dictionaries as global affect list

*IWRAD* Italian Weighted Referential Activity Dictionary; *IRefD* Italian Reflection Dictionary; *IDFD* Italian Disfluency Dictionary; *ISenSD* Italian Sensory-Somatic Dictionary; ISAffD, IPAffD, INAffD, *IZAffD* Italian Dictionary of, respectively, Sum, Positive, Negative, Neutral (Z) Affects

### Statistical analysis

All statistical analyses were performed using the Statistical Package for Social Science version 26 (SPSS version 25). Data is reported as means and standard deviations for continuous variables and as percentages for discrete variables. *T* tests and Chi-square tests were performed to evaluate the homogeneity of the two groups, respectively, for continuous and discrete variables. The *t*-test for paired samples was applied to explore differences between the two groups, using Bonferroni correction for multiple *t*-tests for all measures applied and a Cohen’s D effect size was also performed. A series of logistic binary regressions were performed, using specific linguistic variables as independent variables, which belonged to the clinical or the control group as dependent variables.

## Results

The comparison of the two groups highlighted the following results. With respect to socio-demographic characteristics, the two groups can be considered homogeneous; no significant differences in age and education emerged. In regards to the first hypothesis of the study, the findings which emerged (Table 3) showed that the results for ERQ, TAS-20, and POMS are significantly different (using Bonferroni correction for multiple *t*-test *p* < 0.05).

**Table 3:** Two sample independent t-tests on emotional regulation (ERQ), mood states (POMS), and alexithymia (TAS-20):

	AN Group (N. 29)		Control Group (N. 36)		T (Degrees of freedom 63)	*p*	D-Cohen effect size
	*M*	*Sd*	*M*	*Sd*			
ERQ							
Reappraisal	20.59	5.828	25.53	4.601	-3.821	0.000	-0.841
ERQ Suppression	18.55	5.262	12.56	5.485	4.461	0.000	0.956
POMS—Tension	20.69	7.947	13.89	9.029	3.182	0.002	0.722
POMS—Depression	30.83	13.957	14.42	13.793	4.743	0.000	0.956
POMS – Anger	19.17	11.311	12.78	11.931	2.198	0.032*	0.523
POMS – Vigour	11.17	6.709	17.08	6.959	-3.459	0.001	-0.769
POMS – Fatigue	13.41	7.291	10.33	7.111	1.724	0.090	0.400
POMS – Confusion	16.07	6.771	10.25	5.331	3.878	0.000	0.846
POMS GLOBAL	89.00	46.297	44.58	41.910	4.054	0.000	0.886
TAS-20 DIF	25.38	6.264	15.89	5.908	6.267	0.000	1.233
TAS-20 DDF	19.55	4.641	14.06	5.297	4.391	0.000	0.954
TAS-20 EOT	17.10	3.416	15.72	4.314	1.405	0.165	0.337
TAS-20 TOT	62.03	11.372	45.67	11.556	5.717	0.000	1.158

TAS-20 DIF, Difficulty identifying feelings; TAS-20 DDF, Difficulty describing feelings; TAS-20 EOT, Outward-oriented thinking

^*^not significant results using Bonferroni correction to multiple *t*-test *p* < .05

### Emotional regulation, alexithymia and mood states

This result highlights that participants with anorexia nervosa show greater emotional dysregulation, specifically higher in suppressive strategy, i.e., dysregulated expression of emotions, and lower levels of cognitive reappraisal functional strategies. The AN group displayed significantly higher levels of alexithymia than the control group; however, the mean total score of alexithymia for the AN group overcame the general cut-off > 60, showing a clinical value of alexithymia for the AN group. The sub-factor outward-oriented thinking is the only result that is not significant. The assessment of mood states measured by the POMS shows significant differences in all subfactors except for the fatigue and anger factors.

### Referential process linguistic measures

The second hypothesis explores if specific linguistic features predict the belonging to a clinical or healthy group. The linguistic analysis shows a significantly greater use of sensory-somatic words in the AN group. Regarding the symbolic narrative properties of AN patients, there is a specific difference in the narrative characteristics. AN patients have a significantly higher use of words connoted with negative affect. They also show a significant dysregulation process, resulting in a higher RA intensity index (MH-IWRAD) (see Table 4).

**Table 4:** Two sample independent t-tests on RP the linguistic measures

	*AN group N.29*		*Control Group N.36*		T (Degrees of freedom 63)	*p*	D-Cohen effect size
	*M*	*Sd*	*M*	*Sd*			
IDFD	0.1021	0.0311	0.0921	0.0229	1.484	0.143	0.367
INAffD	0.0156	0.0065	0.0111	0.0041	3.368	0.001	0.779
IPAffD	0.0163	0.0059	0.0131	0.0048	2.383	0.020*	0.573
IZAffD	0.0044	0.0023	0.0045	0.0020	-0.329	0.743	-0.082
ISAffD	0.0364	0.0058	0.0289	0.0070	4.641	0.000	1.007
ISenSD	0.0443	0.0103	0.0354	0.0071	4.115	0.000	0.918
IRefD	0.0320	0.0087	0.0329	0.0095	-0.407	0.685	-0.102
IWRAD	0.5140	0.0033	0.5015	0.0026	1.897	0.042*	0.463
MH-IWRAD	0.0103	0.0022	0.0083	0.0017	2.725	0.008	0.648

IDFD = Italian Disfluency Dictionary; ISAffD, IPAffD, INAffD, IZAffD = Italian Dictionary of, respectively, Sum, Positive, Negative, neutral (Z) Affects; ISenSD = Italian Sensory-Somatic; IRefD = Italian Reflection Dictionary; Dictionary; IWRAD = Italian Weighted Referential Activity Dictionary; MH-IWRAD = Italian Referential Activity Intensity Index;

^*^not significant results using Bonferroni correction to multiple *t* test *p* < .05

To investigate possible predictors of an clinical group, a series of logistic binary regressions were performed using the belonging to the clinical or control group as a dependent variable and linguistic measures, such as Sensory-Somatic, Negative Affects, and the RA Intensity Index as independent variables. The model accounted for 35% (R2 = 0.349; *χ*2 = 27,929; df = 3; *p* = 0.001) of the criterion variable (belonging to the clinical group). In particular, the model showed a significant predictive effect on the AN group of Sensory-Somatic (*β* = 85,099; df = 1; p = 0.039); Negative Affect (*β* = 132,202; df = 1; *p* = 0.011); and Intensity Index (MH-IWRAD; *β* = 322,410; df = 1; *p* = 0.029).

To better explain these findings, we show a clinical vignette from the autobiographical narratives:

### Clinical example: high somatic sense, high negative affect, high RA index

At school the only thing that I will never forget [360,8,15,1], it happened that I had a pseudo panic attack [370,18,15,1], we were doing religion and at a certain [380,28,15,1] point I had kind of a detachment from my head [390,38,15,1], from with the mind and body and practically I have [400,48,15, 1] started screaming in panic, I (I know) felt terrible [410,58,15,1] and I remember that then they called like the ambulance […] I began to stay bad from [450,98,15,1] we were all put in a circle around the desk [490,14,19,1], I don't remember what the teacher was talking about, of [500,24,19,1] which argument, I was listening, then at a certain point [510,34,19,1] I began to feel entire distant voices [520,44,19,1] of the speaker and I remember that I closed my eyes for a moment [530,54,19,1] and then when I opened them again, yes [540,64,19, 1] it was all upside down, that is, everything was spinning, as I no longer felt [550,74,19,1] my body, as if I were no longer inside [560,84,19,1] myself.

## Discussion

The most relevant finding of the study is the association between AN and several features of emotional dysregulation. This aspect is found both in the greater use of suppression strategy, as well as in lower cognitive reappraisal capacity. Moreover, the presence of higher values in all the POMS scales, except for anger and fatigue, suggests greater emotional arousal in almost all of the scales. The elevated mood profile in the AN group highlights a relationship between altered mood states and AN, reinforcing the hypothesis that food control is a form of behavior related to emotional control. In fact, many studies reveal a relationship between AN and mood disorders [55].

Finally, the AN group showed higher levels of alexithymia than the control group, indicating difficulty in recognizing and describing emotions through words. As Taylor & Bagby [14] posed, alexithymia is an effect of a disrupted or failed RP process, i.e., the regulation of emotional arousal, and the construction of emotional meanings [31]. Moreover, activation of the subsymbolic system without symbolic connections may result in poorly regulated states of emotional arousal, which may contribute to the pathogenesis of different disorders, both somatically and psychiatrically [30].

In general, the presence of emotional dysregulation, alexithymia, and altered mood states in AN patients reinforce the centrality of the regulatory function that food assumes as an addiction [56] and also the centrality of an experience of being foreign to one's body [57].

In our study, emotion dysregulation was also indirectly found in the autobiographical narratives, since according to MCT [26], disruptive emotional process can involve the difficulty of symbolizing one's own affective experience. Results confirmed specific linguistic features of the RP in autobiographical narratives of AN patients. Specifically, three linguistic indices were found to be more related to this disorder than others: the sensory-somatic words, the prevalence of negative affect, and the RA Intensity Index (MH-IWRAD), which represents the RP disconnection. The presence of emotional disconnection detected in linguistic measures, together with high indices of sensory-somatic words is in line with the literature linking AN to the use of concrete thinking and metaphors, showing a reduction of symbolic capacity and impaired reflective function [58–60]. Mariani et al. [37] highlighted how language features can be related to a personal expression of psychopathology. The findings of our study demonstrated a relationship between AN and the difficulty of the symbolizing process. Linguistic analysis reveals the presence of concrete and body references that are coherently used to preview findings, suggesting that AN's severe metabolic impairment may have an effect for a peculiar pattern of linguistic difficulties in using the symbolizing process [58, 59].

The clinical vignettes showed specific linguistic features which connected bodily distress, AN symptoms, and personal emotional conflicts. Linguistic analysis emphasizes the relevance in understanding the relationship between words and emotional activation in psychopathology [30, 37, 60, 61].

In conclusion, all these results described a direct relationship between emotion regulation and language, following MCT [29], showing specific patterns of language in autobiographical narrative as an expression of emotional activation. This aspect can be related to possible clinical interventions aimed at narrating one's own experiences, such as the development of the ability to recognize one's affective states during clinical interviews to manage emotional dysregulation [54]. Furthermore, these results support the usefulness of setting up psychotherapeutic treatments aimed at increasing symbolic processes, managing to increase connections between the body, symptoms and mental states [61].

### What is already known on this subject?

AN shows a relevant connection between food control symptoms and difficulty in managing emotions and one's affective states. Maladaptive aspects of emotional regulation and mood alterations were found in connection with anorexia nervosa.

### What does this study add?

This study adds new evidence that AN disorder is closely related to maladaptive strategies utilized to regulate emotions, as well as difficulty in recognizing and symbolizing one's affective states. Furthermore, the analysis of autobiographical narratives strengthens the identification of specific linguistic characteristics of a concrete thought based on the perception of the body and symptoms, with a prevalence of negative affective states.

## Conclusions

The main results of the present study show that the AN group displays higher levels of alexithymia, emotional dysregulation, and mood profile. These results are consistent with recent literature describing AN as a disorder related to emotional regulation and that food control is a possible regulator on which patients depend. This study highlights the importance of investigating the relationship between AN and emotional regulation, especially by trying to explore whether or not patients with AN are lacking an understanding of emotional states of other people, as well as their own [62]. This consideration is particularly important for designing specific treatment interventions.

A further novelty of the study is the exploration of the relationship between linguistic characteristics and AN. It is noted that in the autobiographical stories, AN patients express their illness showing a linguistic index of emotional dysregulation and greater sensory-somatic words and negative affect. This result highlights how language is closely related to the body and its psychic suffering.

## Limitation

Our work explores innovative aspects in the treatment of anorexia nervosa, such as language in relation to emotional regulation. However, it is necessary to highlight the limitations of our study, such as the sample size, which will be progressively increased to confirm the data obtained. Furthermore, it would be useful to be able to better differentiate the processes concerning restrictive AN with other eating disorders. In the future, we also intend to refine the diagnostic evaluation by relating it to other comorbid phenomena and personality disorders, because they, too, can have an impact in the management of acute conditions.

## Data Availability

“The data sets generated and analyzed during the current study are available from the corresponding author upon reasonable request.”

## Abbreviations

ED: Eating Disorders
AN: Anorexia Nervosa
BMI: Body Mass Index
EAT-26: Eating Attitudes Scale Test
SCID-5-CV: Structured Clinical Interview for Dsm-5 Disorders: Clinician Version
MCT: Multiple code theory
TAS-20: Toronto Alexithymia Scale
ERQ: Emotional Regulation Questionnaire
POMS: Profile of Mood of State
RAP: Relationship Anecdotal Paradigm
ER: Emotional Regulation; RP: Referential Process
RA: Referential Activity Intensity Index
IDAAP: Italian Discourse Attributes Analysis Program
IWRAD: Italian Weighted Referential Activity Dictionary
MH-IWRAD: Italian Mean High Weighted Referential Activity Dictionary
IDFD: Italian DisFluency Dictionary
IREF: Italian Reflection Dictionary
ISensD: Italian Sensory Somatic Dictionary
ISAffD: Italian Sum Affect Dictionary
IPAffD: Positive Affect
INAffD: Negative Affect
IZAffD: Neutral Affect
